# Regarding Clinical Management of Polyposis: Do You Mind?

**DOI:** 10.1055/a-2940-7189

**Published:** 2026-09-08

**Authors:** Ilker Sengul, Demet Sengul

**Affiliations:** 1 Thyroidology Unit 485533 Giresun University Faculty of MedicineGiresunTurkey; 2 Division of Endocrine Surgery 485533 Giresun University Faculty of MedicineGiresunTurkey; 3 Department of General Surgery 485533 Giresun University Faculty of MedicineGiresunTurkey; 4 Department of Pathology 485533 Giresun University Faculty of MedicineGiresunTurkey

## 

Dear Editor,

We read with great interest the article by Cui et al. 1 regarding the endoscopic features and management of cap polyposis (CP), published in volume 14, *Endoscopy International Open* . The authors are to be commended for distilling insights from a seven-case series of this rare entity, which remains a formidable diagnostic challenge for even the most seasoned endoscopists. However, several pivotal aspects of the study warrant a more nuanced critical reflection to align these findings with the rigorous standards of high-impact clinical gastroenterology. A primary concern lies in the profound clinical heterogeneity of the cohort. The inclusion of Patient 1, possessing a 13-year history of ulcerative colitis (UC), and Patient 3, with comorbid chronic myelogenous leukemia (CML), introduces significant confounding variables. In such cases, it becomes difficult to discern whether the polypoid lesions represent primary CP or a secondary inflammatory phenomenon exacerbated by underlying systemic dysregulation or long-standing colitis. The “histopathological mimicry” often observed in mucosal prolapse-type lesions requires an even more stringent exclusion of secondary causes to maintain the diagnostic purity of a CP series. Furthermore, the “Helicobacter pylori ( *H. pylori)* 2 3 4 5 hypothesis” presented by the authors is intriguing yet remains mechanistically elusive. While the authors demonstrate clinical improvement in two young patients following eradication, the failure of this therapy in advanced cases—and the absence of *H. pylori* in colonic tissue—suggest that the bacteria may be bystanders rather than direct pathogens. The proposed mechanism involving gut microbiota dysbiosis is compelling, yet without longitudinal microbiome profiling, it remains a post hoc rationalization. We also kindly note the authors’ candid acknowledgment regarding the lack of systematic background mucosal biopsies. In the absence of such data, the differentiation from UC—where microscopic inflammation persists in “normal-appearing” intervening mucosa—rests heavily on macroscopic interpretation. In the context of CP, *H. pylori* may act as a “Trojan Horse”; its eradication appears to conquer the disease in some, yet the true “warrior” causing the colonic inflammation remains hidden behind the walls of an unidentified systemic or microbial trigger. To reach the zenith of diagnostic precision, future studies must mandate standardized biopsy protocols of nonpolypoid areas to definitively rule out diffuse inflammatory bowel disease. In essence, while this study illuminates the varied clinical trajectories of CP, it reinforces the need to transition from retrospective observations to prospective, multicenter registries. Until a standardized therapeutic algorithm is validated, the management of CP will remain a bespoke endeavor rather than a codified clinical practice. This issue merits further investigation. We thank Cui *et al* . 1 for their valuable study in *Endosc Int Open* on clinical management of polyposis.

## Data Availability

The datasets used and/or analyzed during this study are available from the corresponding author upon reasonable request.
